# Daytime napping, sleep duration and increased 8-year risk of type 2 diabetes in a British population

**DOI:** 10.1016/j.numecd.2016.06.006

**Published:** 2016-11

**Authors:** Y. Leng, F.P. Cappuccio, P.G. Surtees, R. Luben, C. Brayne, K.-T. Khaw

**Affiliations:** a Institute of Public Health, University of Cambridge, UK; b Division of Mental Health & Wellbeing, Warwick Medical School, University of Warwick, Coventry, UK

**Keywords:** Sleep, Type 2 diabetes, Epidemiology, Prospective, Cohort

## Abstract

**Background and aims:**

Few studies have prospectively examined the relationship between daytime napping and risk of type 2 diabetes. We aimed to study the effects of daytime napping and the joint effects of napping and sleep duration in predicting type 2 diabetes risk in a middle- to older-aged British population.

**Methods and results:**

In 1998–2000, 13 465 individuals with no known diabetes participating in the European Prospective Investigation into Cancer-Norfolk study reported daytime napping habit and 24-h sleep duration. Incident type 2 diabetes cases were identified through multiple data sources until 31 July 2006. After adjustment for age and sex, daytime napping was associated with a 58% higher diabetes risk. Further adjustment for education, marital status, smoking, alcohol intake, physical activity, comorbidities and hypnotic drug use had little influence on the association, but additional adjustment for BMI and Waist Circumference attenuated the Odds ratio (OR) (95% CI) to 1.30 (1.01, 1.69). The adjusted ORs (95% CI) associated with short and long sleep duration were 1.46 (1.10, 1.90) and 1.64 (1.16, 2.32), respectively. When sleep duration and daytime napping were examined together, the risk of developing diabetes more than doubled for those who took day naps and had less than 6 h of sleep, compared to those who did not nap and had 6–8 h of sleep.

**Conclusion:**

Daytime napping was associated with an increased risk of type 2 diabetes, particularly when combined with short sleep duration. Further physiological studies are needed to confirm the interaction between different domains of sleep in relation to diabetes risk.

## Highlights

• Daytime napping was independently associated with a 30% increase in diabetes risk.
• The association was partly explained by adiposity.
• Short and long sleep was associated with a 46% and 64% increase in diabetes risk.
• The risk of diabetes more than doubled for those taking day naps and sleep for < 6 h.

## Introduction

The association between sleep and diabetes has attracted extensive attention in the last decade [1]. Sleep duration has been the centre of the discussion, with several meta-analysis confirming a U-shaped relationship between sleep duration and risk of type 2 diabetes [2], [3]. More recently, a potential link between daytime napping and type 2 diabetes has been reported. Cross-sectional studies have suggested that napping was associated with an increased prevalence of diabetes or impaired fasting plasma glucose in Chinese older adults [4], [5], and in White postmenopausal women [6]. This raises questions as to whether daytime napping could simply be a consequence of diabetes. Indeed, the Health ABC study showed earlier in 235 older adults that the odds of day napping, as recorded by wrist actigraphs, was five times higher in individuals with diabetes compared to those without [7]. As such, prospective study designs may help determine the nature of this relationship.

To date only two published prospective studies with diabetes as the primary outcome have attempted to address this issue. One study concluded that day napping may be an independent risk factor for self-reported incident diabetes in an American population [8]. The association was modestly attenuated by adjustment for Body Mass Index (BMI), and was stronger among those with short nighttime sleep durations. A more recent study [9] found a non-significant association between frequent napping and incident type 2 diabetes in a middle-aged Finnish population, after accounting for BMI. These intriguing findings, particularly the joint effects of daytime and nighttime sleep and the influence of obesity need to be confirmed by larger prospective studies with validated ascertainment of diabetes cases, and with consideration of a wider range of anthropometric measures. Moreover, since daytime napping has a strong cultural implication, it is important to compare findings in different cultural settings.

We set out to study the prospective association between daytime napping and the risk of type 2 diabetes in a middle- to older-aged British population. Specifically this study aimed to test the role of anthropometric measures, and whether there is an interaction between daytime napping and sleep duration in relation to diabetes risk.

## Methods

### Study population

Participants were drawn from the European Prospective Investigation of Cancer-Norfolk (EPIC-Norfolk) cohort study, which is part of an ongoing multi-centre prospective cohort study [10]. Details of the design of the study have been described previously [11]. Briefly, a total of 25 639 men and women aged 40–74 years attended the baseline health check during 1993–1997, and were then followed up for two further health checks from 1996 to 2000 and from 2006 to 2011. In between these health examinations, participants were sent various questionnaires, which contained questions on sleep characteristics. The Norwich District Ethics Committee approved the study and all participants gave signed informed consent.

### Measures

Participants were asked to report their daytime napping habit during 1998–2000. This information was collected through a yes-no question “Do you normally take a nap during the day?” In addition, participants were asked “On average, how many hours do you sleep in a 24 h period?” with six response options: “<4, 4–6, 6–8, 8–10, 10–12 and >12”.

All covariates were chosen a-priori based on their relevance to sleep and diabetes [12], [13], [14]. Socio-demographic information were obtained through the baseline health questionnaire, and included age, sex, educational (highest qualification attained: no qualifications, educated to age 16 years, educated to age 18 years, or educated to degree level), and marital status (single, married, widowed, separated, or divorced). The follow up health questionnaire collected information on smoking status (current, former, or non-smokers), alcohol intake (units of alcohol drunk per week) and hypnotic drug use (yes/no). Physical activity levels was reported in four categories (inactive, moderately inactive, moderately active, or active) through a validated questionnaire [15]. Comorbidities included self-reported cardiovascular diseases (CVD), cancer, asthma and bronchitis. Anthropometric measures included objective assessed BMI (weight in kilograms divided by height in meters squared) and waist circumference (WC). WC was defined as the smallest circumference between the ribs and iliac crest, or at the level of the umbilicus if there was no natural waistline, and was measured to the nearest 0.1 cm with the volunteers standing with abdomen relaxed at the end of a normal expiration.

Participants who reported doctor-diagnosed diabetes or diabetes-specific medication use at the time of sleep measurement were excluded from the analysis (n = 629). The final study sample included 13 465 participants with complete information on daytime napping and the covariates. Incident type 2 diabetes cases were identified through multiple data sources [16] until 31 July 2006. These included self-reported doctor-diagnosed diabetes cases and diabetes-specific medication use, verified through record linkage with the general practice diabetes register, local hospital diabetes register, hospital admissions data and Office of National Statistics death certificate data with coding for diabetes. A new case was defined by a doctor's diagnosis of type 2 diabetes with no insulin prescribed within the first year following diagnosis and/or a glycated haemoglobin (HbA1c) level of greater than 7 percent at the health check. Self-reported diabetes cases that could not be verified were not included as incident cases.

### Statistical analysis

Baseline characteristics of the participants were firstly compared by daytime napping (yes/no), using Pearson's χ^2^ test for categorical variables and student's t-test for continuous variables. As the influence of sex on BMI and WC is potentially important, the sex-specific mean (SD) of these two measures were presented. Multivariable logistic regression was used to obtain OR (95% CI) for incident diabetes by daytime napping (with no napping being the reference group), and by sleep duration (with 6–8 h being the reference group, due to the U-shaped relationship reported previously [3]). Three models were fitted with progressive adjustment of the covariates: Model A adjusted for age and sex; model B further adjusted for education, marital status, smoking status, alcohol intake, physical activity, comorbidities and hypnotic drug use; model C additionally accounted for BMI and WC.

Subgroup analysis was conducted to explore the effects of potential effect modifiers, including age (<65 vs. > = 65 years old), sex, social class (non-manual vs. manual), BMI, WC, pre-existing diseases and self-reported general health. The interaction between day napping and sleep duration was also tested, and the joint effects for different combinations of napping and sleep duration categories were presented, using participants with no napping and 6–8 h of daily sleep duration as the reference group. All subgroup analysis was conducted using model B to avoid over-adjustment by anthropometric measures. All statistical tests were 2-sided, and a p value <0.05 was considered statistically significant. Analyses were implemented in Stata, version 12.0 (StataCorp LP, College Station, Texas).

## Results

In total, 3852 (28.6%) of the participants reported day napping, and 9285 (67.9%) of them reported sleeping for 6–8 h per day. Table 1 shows baseline characteristics of the participants by napping habit. Those who reported napping were older, more likely to be men and to have lower education. They were also more likely to be current or former smokers, physically inactive, to be taking hypnotic drugs and to have comorbidities. Supplemental Table 1 summarises the sex-specific distributions of BMI and WC by sleep. Both men and women who reported daytime napping had a higher BMI and higher WC. Those who reported short sleep duration also had a slightly higher BMI and WC, although the overall difference was not statistically significant in women.

Table 2 shows the adjusted OR (95% CI) of diabetes incidence by napping and 24-hour sleep duration. After adjustment for age and sex, daytime napping was associated with a more than 50% higher diabetes risk. Further multivariable adjustment had little influence on the association, but additional adjustment for BMI and WC attenuated the OR (95% CI) to 1.30 (1.01, 1.69). For sleep duration, adjustment for BMI and WC mainly attenuated the association for short sleep duration. After adjustment for all covariates, the ORs (95% CI) associated with short and long sleep duration were 1.46 (1.10, 1.90) and 1.64 (1.16, 2.32), respectively.

Results from subgroup analysis are summarised in Supplemental Tables 2 and 3. The association between daytime napping and diabetes incidence appeared to be stronger among those with a higher WC (OR = 1.54, 95% CI: 1.17, 2.03) and those reporting poorer general health (OR = 1.75, 95% CI: 1.06, 2.89), although neither of these tests for interaction was significant. The associations for sleep duration across subgroups showed a similar pattern, with no significant interaction observed between sleep duration and any of the stratified variables.

**Figure 1:**
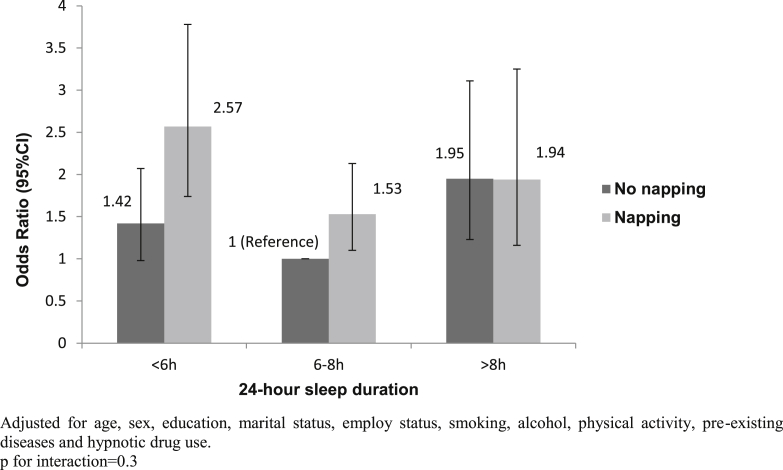
Joint effects of daytime napping and sleep duration on the risk of type 2 diabetes. Adjusted for age, sex, education, marital status, employ status, smoking, alcohol, physical activity, pre-existing diseases and hypnotic drug use. p for interaction = 0.3.

Fig. 1 shows the joint effects of daytime napping and sleep duration on diabetes incidence. The risk of diabetes was lowest among those who did not nap and had 6–8 h of sleep. The association between napping and diabetes risk was not seen among those with more than 8 h of sleep. Long sleep was associated with more than 90% increased risk of diabetes, regardless of the napping behaviour. However, the association between short sleep and diabetes risk was much stronger among those who reported napping. The OR (95% CI) for persons who both had <6 h of sleep and reported napping was 2.57 (1.74, 3.78). The overall interaction between sleep duration and daytime napping did not reach statistical significance (p = 0.3).

## Discussion

This prospective cohort study suggested that daytime napping was independently associated with an increased diabetes risk in a middle- to older-aged British population. The association was partly explained by anthropometric measures, but not entirely with a 30% increased diabetes risk associated with napping after controlling for BMI and WC, in addition to adjustment for socio-demographic and lifestyle factors. A U-shaped relationship was observed between sleep duration and diabetes risk. After adjustment for all covariates, short sleep duration and long sleep duration was associated with a 46% and 64% increase in diabetes risk, respectively. The risk of developing diabetes more than doubled for those who took day naps and had less than 6 h of sleep, compared to those who did not nap and had 6–8 h of sleep per day.

This is the first study to examine the relationship between daytime napping, sleep duration and diabetes risk in a British population. The study benefited from a prospective design, validated ascertainment of diabetes cases in a large population sample, and objectively measured anthropometric measures including both BMI and WC to help understand the association. Some limitations need to be considered. First, the analysis was restricted to participants without diabetes at baseline and with complete measures of covariates. Compared to the rest of the baseline sample, these participants were younger, more likely to be women, and more likely to be of higher social class and higher education. While this might have influenced the external validity of the study, selection bias is unlikely as the incidence of diabetes was similar between those included and excluded from the current analysis. Second, diabetes cases were identified and verified primarily by health records, and some of the underlying cases could have been missed. However, HbA1c was also used as a criteria to help address undiagnosed cases, and it was reported previously in this cohort that the number of unidentified cases was relatively small and was unlikely to have led to significant bias [16]. Therefore, the case identification approach used in this study should provide a relatively accurate estimate, especially given that multiple oral glucose tolerance testings on all individuals might not be feasible for large population-based studies. In addition, daytime napping and sleep duration were reported through questionnaires, which might have introduced measurement errors and the true association could have been attenuated. Reported sleep reflects individual's own perception and could be subject to selection bias and recall bias. In order to retain power, daytime napping was dichotomised, without further categorization of napping durations. Sleep duration was grouped into three categories based on a U-shaped relationship found by most previous studies [2]. Therefore, we were unable to differentiate the effects of power naps from those of excessive napping, and could not investigate the differences associated with each hour of change in sleep duration. While this might have diluted the overall association, these two reported sleep measures have been previously associated with other health endpoints in the EPIC-Norfolk cohort [17], [18]. From a practical standpoint, the perception of general sleep habits should have validity in itself and is also a more feasible measure in primary care settings. Future studies need to examine in more detail the effects of different napping durations as well as more extreme sleep durations. Accelerometry recording over a prolonged period of participants' daytime and nighttime sleep might also help to resolve the problem. Finally, obstructive sleep apnea (OSA) and poor sleep quality have been associated with both napping and diabetes [1], [19], [20], and might have confounded the association. In order to address this problem, we adjusted for adiposity, an important marker of OSA and respiratory impairment [21], [22]. However, residual confounding might still exist, and future studies should include assessment of OSA and sleep quality to help understand this relationship.

Consistent with previous findings [4], [8], this prospective study found an independent association between daytime napping and increased diabetes risk. Several cross-sectional studies have been conducted among Chinese older adults, and have found a dose–response relationship between the duration of napping and increased prevalence of diabetes, after adjustment for either BMI or WC [4], [5]. One recent study found in a Chinese population that long sleep duration and long daytime napping were independently and jointly associated with higher diabetes risk [23]. Unlike in the UK, daytime napping in China is a social norm and is practiced by many people as a routine. Therefore, the implications of napping in the British could be different from that in the Chinese, where only long and frequent naps might be related to adverse health outcomes. While the lengths of naps were not noted in the current study, we found an increased diabetes risk associated with napping habit overall, regardless of the duration of the naps. Indeed, in a population who do not normally take naps, the presence of napping habit could have potential indicative implications for health. Notably, the nappers were generally older and had a less favourable health profile, so the possibility of residual confounding by declining health cannot be ruled out. However, napping habit was reported years before diabetes incidence, and the association was independent of pre-existing diseases and did not differ by self-reported general health, making this explanation unlikely on its own.

The role of obesity in the association between daytime napping and diabetes risk is particularly interesting. Both of the two prospective studies have tried to address this issue and have found the association attenuated after accounting for BMI [8], [9]. Xu et al. reported that the associations remained significant in a US national sample of older adults but proposed that a more precise measurement of adiposity might further attenuate it [8], while the most recent study among Finnish middle-aged adults suggested complete attenuation of the association [9]. This could be due to the smaller sample size of the latter study, or could be because of different pathways for people of different ages. The current study included both BMI and WC as covariates, and performed subgroup analyses to test if the association differed by BMI or WC. While we showed that those who took naps indeed had higher BMI and WC and adjustment for these adiposity measures attenuated the association between napping and diabetes risk, there remained a 30% significantly increased risk after all the adjustment. This indicated that adiposity might explain part but not all of the association between napping and diabetes in this population. Notably, additional adjustment for adiposity measures mainly attenuated the association for short sleep rather than for long sleep duration. It is possible that obesity explains more of the association between short sleep duration and diabetes risk in this population. Subgroup analysis suggested that the association between napping and diabetes was only significant among those with a higher WC, although test for interaction was not statistically significant. Larger studies should further investigate whether daytime napping is linked to diabetes only in obese populations or if this should be noted generally in middle- to older-aged adults.

Interestingly, participants who took daytime naps but slept for short hours at night had the highest diabetes risk. This is in keeping with finding from an earlier US study [8]. Meanwhile, the differences in diabetes risk by napping habit cannot be observed among those with long sleep duration. It is plausible that short sleep duration and daytime napping were both indicators of sleep disturbance or share a common pathway, leading to an additive combined effect of the two factors. The mechanisms for long sleep and diabetes risk remain to be elucidated, with residual confounding and reverse causality being major concerns [3]. It should be noted that even among those with sleep duration of 6–8 h, daytime napping was still associated with a 53% increase in diabetes risk. The test for interaction between daytime napping and sleep duration, however, was not significant in this study. This might be due to a lack of power to detect interaction, and larger studies are needed in the future to help understand this potential interaction effect.

Although daytime napping was traditionally considered as a healthy lifestyle [24], it has been increasingly associated with adverse health outcomes in the past few years [25], [26]. A number of recent meta-analysis consistently reported an increased mortality risk associated with daytime napping [27], [28], yet the underlying health implications of daytime napping remain to be examined. The biological mechanisms triggered by day naps are unknown, and it is unclear which system is most directly involved with napping that eventually links to an increased mortality risk. The accumulating evidence on daytime napping and increased risk of type 2 diabetes suggested the need for a closer look at the potential mechanisms. In the EPIC-Norfolk cohort, daytime napping was previously associated with an increased all-cause mortality risk [17] and a higher level of pro-inflammatory marker [29]. Since elevated levels of inflammatory biomarkers have been associated with sleep, sedentary time and an increased risk of diabetes [30], [31], [32], this could be a plausible pathway. In addition, increased sympathetic activity upon awaking from daytime naps, especially prolonged naps, could lead to disruption of the sympatho-vagal balance, activation of the renin-angiotensin system, and subsequently modulated insulin secretion and glycaemic control [33], [34]. One study found increased evening cortisol levels associated with excessive daytime napping in nursing home residents with dementia [35]. Besides, an experimental study has suggested a 16% rise in glucose levels, 55% increase in insulin secretion, and 39% increase in serum insulin levels, associated with daytime sleep [36]. Notably, this study focused on circadian rhythmicity and thereby involved 8 h of daytime sleep, which seems unrealistic in a natural setting. Another population-based cohort study found that napping >30 min was associated with a higher risk of abnormal glucose metabolism [37]. While the exact biological mechanisms for the association between sleep habits and diabetes requires further exploration, it is possible that other genetic and environmental factors have played an role, and thus causal relationship cannot be established. Notably, the protective effects of daytime napping, known as the ‘siesta’, have been reported mostly in Mediterranean settings a long time ago [24], whereas findings from northern European countries or the US [6], [9], [25] often contrast with these traditional views. In countries where daytime napping is not a routine, it is likely that we are describing a ‘forced’ napping behaviour driven both by pre-existing conditions and by insufficient nightitme sleep that makes the daytime napping catchup more common. Furthermore, the attenuation of the risk after adjustment for BMI and WC also suggests OSA as a potential mediator, given the close relationship between obesity and OSA [38]. OSA might lead to shortened and disrupted nighttime sleep, increased daytime sleepiness, and has been independently associated with diabetes risk [19], [20], further supporting our observation on the interaction between short sleep and daytime napping in predicting diabetes risk. On the whole, adiposity as a potentially important mediating factor requires further exploration, especially given the existing evidence on napping and increased odds of obesity [39]. Studies incorporating measures of neck circumference and OSA might help to address this question in the future.

In summary, this prospective cohort study suggested that daytime napping was independently associated with a 30% increased diabetes risk in a British population. The relationship between daytime napping and diabetes risk was more pronounced among those with short sleep durations; the combined risk of developing diabetes for those who took day naps and had less than 6 h of sleep more than doubled compared to those with 6–8 h of sleep and without day naps. While adiposity partly explains the associations, further studies are needed to help understand the nature of the relationship and the joint effects of daytime napping and sleep duration in predicting future risk of type 2 diabetes.

## Authors' contributions

The work presented here was carried out in collaboration between all authors. YL analysed these data and wrote the manuscript with co-authors. YL, FPC, PGS, CB and KTK discussed the analysis, interpretation and presentation of these data. RL performed all data management and record linkage. RL and KTK are on the management team of EPIC-Norfolk population study and contributed substantially to acquisition of data. KTK is a principal investigator in the EPIC-Norfolk study. All authors provided detailed comments on the draft, and revised the manuscript critically. All authors read and approved the final manuscript.

## Guarantor

Dr. Yue Leng.

## Funding

The design and conduct of the EPIC-Norfolk study and collection and management of the data was supported by programme grants from the Medical Research Council UK (G9502233, G0300128) and Cancer Research UK (C865/A2883). Funding sources did not have a role in the design and conduct of the study; collection, management, analysis, and interpretation of the data; and preparation, review, or approval of the manuscript.

## Conflict of interest

The authors declared no conflict of interest.

## Figures and Tables

**Table:** Number of participants with (and without) diabetes by categories of sleep duration and napping.

Sleep duration	Napping	
	No	Yes
<6 h	42 (1980)	41 (846)
6–8 h	93 (6556)	67 (2337)
>8 h	23 (829)	19 (506)

**Table 1:** Baseline characteristics of the participants according to daytime napping.

	Daytime napping, no. (%)		p-Value
	No	Yes	
	9613 (71.4)	3852 (28.6)	
**Age**			
<65	6794 (70.7)	1631 (42.3)	<0.001
≥65	2819 (29.3)	2221 (57.7)	
**Sex**			
Men	3789 (39.4)	2098 (54.5)	<0.001
Women	5824 (60.6)	1754 (45.5)	
**Education**			
Lower	4019 (41.8)	1819 (47.2)	<0.001
Higher	5594 (58.2)	2033 (52.8)	
**Marital status**			
Single	375 (3.9)	161 (4.2)	
Married	7795 (81.1)	3070 (79.7)	
Others^a^	1443 (15.0)	621 (16.1)	
**Smoking status**			
Current smokers	764 (7.9)	329 (8.5)	<0.001
Former smokers	3841 (40.0)	1873 (48.6)	
Never smoked	5008 (52.1)	1650 (42.8)	
**Alcohol intake**^b^			
≤3.5	4850 (50.5)	1975 (51.3)	
>3.5	4763 (49.5)	1877 (48.7)	
**Physical activity**			
Inactive	2287 (23.8)	1223 (31.7)	<0.001
Moderately inactive	2938 (30.6)	1052 (27.3)	
Moderately active	2473 (25.7)	846 (22.0)	
Active	1915 (19.9)	731 (19.0)	
**Hypnotic drug use**			
No	9458 (98.4)	3762 (97.7)	<0.01
Yes	155 (1.6)	90 (2.3)	
**Pre-existing diseases**			
No	7188 (74.8)	2656 (69.0)	<0.001
Yes	2425 (25.2)	1196 (31.0)	

a Widowed, separated or divorced.

b Units/week, divided by median.

**Table 2:** Odds ratios (95% CI) of diabetes by daytime napping and 24-h sleep duration.

Sleep (no. of cases)	Total no.	Model A	Model B	Model C
Napping (n = 287)	13 464			
No	9613	Reference	Reference	Reference
Yes	3851	*1.58 [1.23,2.03]	*1.56 [1.21,2.00]	^‡^1.30 [1.01,1.69]
Sleep duration (n = 294)	13 679			
6–8 h	9284	Reference	Reference	Reference
<6 h	2993	*1.65 [1.26,2.16]	^†^1.54 [1.18,2.03]	^†^1.46 [1.10,1.90]
>8 h	1402	^†^1.70 [1.21,2.39]	^†^1.68 [1.20,2.36]	^†^1.64 [1.16,2.32]

Values are presented as Odds ratios (95% CI).

Model A adjusted for age and sex; model B adjusted for age, sex, education, marital status, smoking, alcohol intake, physical activity, pre-existing diseases and hypnotic drug use; model C further adjusted for body mass index and waist circumference.

*p < 0.001; ^†^p < 0.01; ^‡^p < 0.05.
