# Supplementary material for: Daytime napping, sleep duration and increased 8-year risk of type 2 diabetes in a British population
Source: Nutr Metab Cardiovasc Dis. 2016 Nov;26(11):996–1003. doi: 10.1016/j.numecd.2016.06.006 (PMC5084679; doi:10.1016/j.numecd.2016.06.006)
Supplement: Supplementary file 1 [file mmc1.docx]

| Supplementary table 1 Sex-specific BMI and WC according to daytime napping and 24-h sleep duration | | | | | |
| --- | --- | --- | --- | --- | --- |
|  |  | **BMI (kg/m^2^)** | | **WC (cm)** | |
| **Men** | No. | mean | SD | mean | SD |
| Napping |  |  |  |  |  |
| No | 3789 | *26.6 | 3.1 | *94.9 | 9.1 |
| Yes | 2098 | 27.1 | 3.4 | 97.6 | 10 |
| 24-h Sleep duration |  |  |  |  |  |
| <6h | 1092 | *27.0 | 3.5 | †96.6 | 10 |
| 6-8h | 4280 | 26.7 | 3.2 | 95.6 | 9.3 |
| >8h | 604 | 26.8 | 3.4 | 96.5 | 10 |
| **Women** | No. | mean | SD | mean | SD |
| Napping |  |  |  |  |  |
| No | 5824 | *26.2 | 4.2 | *81.0 | 10.4 |
| Yes | 1754 | 27.1 | 4.5 | 84.0 | 10.9 |
| 24-h Sleep duration |  |  |  |  |  |
| <6h | 1901 | 26.7 | 4.4 | 82.4 | 10.7 |
| 6-8h | 5005 | 26.4 | 4.3 | 81.4 | 10.5 |
| >8h | 798 | 26.4 | 4.3 | 81.7 | 10.4 |
| BMI, Body Mass Index; WC, Waist Circumference.  * p <0.001; † p<0.01. | | | | | |

| Supplementary table 2 Subgroup analysis on daytime napping and diabetes incidence | | | | |
| --- | --- | --- | --- | --- |
|  |  |  | Napping | |
|  | Total No. | No. of cases | OR^*^ | 95%CI |
|  |  |  |  |  |
| **Age** |  |  |  |  |
| <65y | 7979 | 128 | 1.46 | [0.98,2.16] |
| >=65y | 5485 | 159 | ‡1.64 | [1.18,2.28] |
| p for interaction |  |  | 0.91 |  |
| **Sex** |  |  |  |  |
| Men | 5886 | 168 | ‡1.55 | [1.12,2.16] |
| Women | 7578 | 119 | †1.54 | [1.04,2.29] |
| p for interaction |  |  | 0.97 |  |
| **Social class** |  |  |  |  |
| Non-manual | 8474 | 157 | 1.35 | [0.95,1.91] |
| Manual | 4769 | 129 | ‡1.77 | [1.22,2.57] |
| p for interaction |  |  | 0.45 |  |
| **BMI (kg/m^2^)** |  |  |  |  |
| <26.1 | 6741 | 54 | 1.48 | [0.82,2.64] |
| ≥26.1 | 6723 | 233 | ‡1.48 | [1.12,1.96] |
| p for interaction |  |  | 0.89 |  |
| **WC (cm)** |  |  |  |  |
| 56.4-87.6 | 6741 | 49 | 1.13 | [0.59, 2.16] |
| 87.7-152.2 | 6723 | 238 | ‡1.54 | [1.17, 2.03] |
| p for interaction |  |  | 0.5 |  |
| **Pre-existing diseases** |  |  |  |  |
| Yes | 3620 | 87 | 1.29 | [0.81,2.05] |
| No | 9844 | 200 | §1.70 | [1.26,2.30] |
| p for interaction |  |  | 0.22 |  |
| **Self-reported general health** | |  |  |  |
| Good to excellent | 11230 | 217 | †1.45 | [1.08, 1.94] |
| Poor to moderate | 2163 | 70 | †1.75 | [1.06, 2.89] |
| p for interaction |  |  | 0.79 |  |
| BMI, Body Mass Index; WC, Waist Circumference.  * adjusted for age, sex, education, marital status, employ status, smoking, alcohol, physical activity, pre-existing diseases, hypnotic drug use; with ‘no napping’ being the reference group.  † <0.05; ‡ <0.01; § <0.001 | | | | |

| Supplementary table 3 Subgroup analysis on sleep duration and diabetes incidence | | | | | | | |
| --- | --- | --- | --- | --- | --- | --- | --- |
|  |  |  | <6h |  | 6-8h | >8h |  |
|  | Total No. | No. of cases | OR^*^ | 95%CI |  | OR^*^ | 95%CI |
| **Age** |  |  |  |  |  |  |  |
| <65y | 8098 | 133 | 1.43 | [0.94,2.15] | 1.00 | ‡1.98 | [1.20,3.28] |
| >=65y | 5581 | 161 | ‡1.67 | [1.16,2.40] | 1.00 | 1.48 | [0.93,2.36] |
| p for interaction |  |  | 0.89 |  |  |  |  |
| **Sex** |  |  |  |  |  |  |  |
| Men | 5975 | 174 | †1.49 | [1.03,2.16] | 1.00 | ‡1.81 | [1.18,2.77] |
| Women | 7704 | 120 | †1.60 | [1.07,2.39] | 1.00 | 1.53 | [0.86,2.71] |
| p for interaction |  |  | 0.85 |  |  |  |  |
| **Social class** |  |  |  |  |  |  |  |
| Non-manual | 8595 | 163 | †1.48 | [1.02, 2.15] | 1.00 | †1.73 | [1.11, 2.70] |
| Manual | 4864 | 131 | †1.61 | [1.08, 2.40] | 1.00 | 1.67 | [0.98, 2.85] |
| p for interaction |  |  | 0.91 |  |  |  |  |
| **BMI (kg/m^2^)** |  |  |  |  |  |  |  |
| <26.1 | 6838 | 55 | ‡2.37 | [1.32,4.23] | 1.00 | 1.6 | [0.69,3.72] |
| ≥26.1 | 6841 | 239 | 1.35 | [0.99,1.84] | 1.00 | ‡1.65 | [1.13,2.40] |
| p for interaction |  |  | 0.18 |  |  |  |  |
| **WC (cm)** |  |  |  |  |  |  |  |
| <26.1 | 6838 | 51 | 1.36 | [0.73, 2.53] | 1.00 | 1.00 | [0.39, 2.60] |
| ≥26.1 | 6841 | 243 | ‡1.56 | [1.15, 2.11] | 1.00 | ‡1.87 | [1.29, 2.70] |
| p for interaction |  |  | 0.54 |  |  |  |  |
| **Pre-existing diseases** | |  |  |  |  |  |  |
| No | 10025 | 204 | †1.50 | [1.08,2.08] | 1.00 | †1.59 | [1.05,2.40] |
| Yes | 3654 | 90 | †1.69 | [1.04,2.74] | 1.00 | †1.86 | [1.02,3.39] |
| p for interaction |  |  | 0.91 |  |  |  |  |
| **Self-reported general health** | |  |  |  |  |  |  |
| Good to excellent | 11403 | 220 | 1.33 | [0.95,1.85] | 1.00 | †1.53 | [1.03,2.26] |
| Poor to moderate | 2197 | 74 | †1.81 | [1.08,3.06] | 1.00 | †2.17 | [1.07,4.39] |
| p for interaction |  |  | 0.48 |  |  |  |  |
| BMI, Body Mass Index; WC, Waist Circumference.  *. adjusted for age, sex, education, marital status, employ status, smoking, alcohol, physical activity, pre-existing diseases, hypnotic drug use.  † <0.05; ‡ <0.01 | | | | | | | |
